# Motor tics evoked by striatal disinhibition in the rat

**DOI:** 10.3389/fnsys.2013.00050

**Published:** 2013-09-18

**Authors:** Maya Bronfeld, Dorin Yael, Katya Belelovsky, Izhar Bar-Gad

**Affiliations:** The Leslie and Susan Gonda (Goldschmied) Multidisciplinary Brain Research Center, Bar-Ilan UniversityRamat-Gan, Israel

**Keywords:** basal ganglia, Tourette syndrome, striatum, bicuculline, GABA

## Abstract

Motor tics are sudden, brief, repetitive movements that constitute the main symptom of Tourette syndrome (TS). Multiple lines of evidence suggest the involvement of the cortico-basal ganglia system, and in particular the basal ganglia input structure—the striatum in tic formation. The striatum receives somatotopically organized cortical projections and contains an internal GABAergic network of interneurons and projection neurons' collaterals. Disruption of local striatal GABAergic connectivity has been associated with TS and was found to induce abnormal movements in model animals. We have previously described the behavioral and neurophysiological characteristics of motor tics induced in monkeys by local striatal microinjections of the GABA_A_ antagonist bicuculline. In the current study we explored the abnormal movements induced by a similar manipulation in freely moving rats. We targeted microinjections to different parts of the dorsal striatum, and examined the effects of this manipulation on the induced tic properties, such as latency, duration, and somatic localization. Tics induced by striatal disinhibition in monkeys and rats shared multiple properties: tics began within several minutes after microinjection, were expressed solely in the contralateral side, and waxed and waned around a mean inter-tic interval of 1–4 s. A clear somatotopic organization was observed only in rats, where injections to the anterior or posterior striatum led to tics in the forelimb or hindlimb areas, respectively. These results suggest that striatal disinhibition in the rat may be used to model motor tics such as observed in TS. Establishing this reliable and accessible animal model could facilitate the study of the neural mechanisms underlying motor tics, and the testing of potential therapies for tic disorders.

## Introduction

Tourette syndrome (TS) is a childhood-onset neurological disorder characterized by the persistent expression of motor and vocal tics (American Psychiatric Association, 2000). Tics are involuntary, repetitive stereotyped movements or vocalizations. Their severity and complexity vary in the range between “simple tics,” which involve only one or a few closely related muscle groups whose activation leads to isolated brief jerk-like movements, and “complex tics,” which involve sequential or coordinated activation of several muscle groups. Although the neuronal mechanisms underlying motor tics remain unknown, multiple studies suggest the involvement of the cortico-basal ganglia (CBG) loop, and specifically the striatum, in the pathophysiological processes leading to tics (Peterson et al., 1998, 2003; Albin et al., 2003; Bloch et al., 2005; Kalanithi et al., 2005; Wang et al., 2011; Worbe et al., 2012).

The basal ganglia (BG) are a group of interconnected sub-cortical nuclei, which form a feedback loop with the cortex. The BG are involved in motor, associative and limbic processes (Alexander et al., 1986), and their dysfunction has been implicated in multiple motor and psychiatric disorders, including Parkinson's disease (Deuschl et al., 2000), Huntington's disease (Reiner et al., 1988), obsessive compulsive disorder (OCD) (Modell et al., 1989) and TS (Singer and Minzer, 2003; Abelson et al., 2005; Kalanithi et al., 2005). The striatum is the main input structure of the BG which receives glutamatergic inputs from the cerebral cortex and the thalamus and dopaminergic inputs from the midbrain (Kemp and Powell, 1971; Wilson et al., 1983; Haber et al., 2000). The striatum projects internally to other BG nuclei which ultimately influence cortical activity via their innervation of the thalamus (Albin et al., 1989; DeLong, 1990). The striatum can be sub-divided into motor, associative, and limbic domains, defined by the origins of the cortical inputs to these territories (Alexander et al., 1986). In the rat, the striatum can be roughly divided into the sensorimotor dorso-lateral part (Cospito and Kultas-Ilinsky, 1981; Ebrahimi et al., 1992), the associative dorso-medial part (Reep et al., 2003), and the limbic ventral territory (Berendse et al., 1992). The dorso-lateral striatum receives somatotopically organized sensorimotor inputs from the cortex, but studies differ as to the exact location, organization and segregation of the somatic territories within the striatum (Webster, 1961; Ebrahimi et al., 1992; Brown and Sharp, 1995; Brown et al., 1998).

The striatal cell population is composed of a single population of projection neurons and multiple types of interneurons. The vast majority of neurons (estimated to be 95% in the rat) are the GABAergic projection neurons, also known as medium spiny neurons (MSNs) (Kemp and Powell, 1971). The striatum also includes multiple types of GABAergic interneurons, such as the fast spiking interneurons (FSIs), low threshold spiking neurons (LTS), and others (Koos and Tepper, 1999; Tepper and Bolam, 2004). Thus, GABA is a key neurotransmitter of the intrastriatal network as it mediates inputs from interneurons, MSN collaterals (Wilson and Groves, 1980; Bolam et al., 2000) and external input from other parts of the BG (namely, the globus pallidus externus—GPe) (Mallet et al., 2012).

Dysfunction of GABAergic transmission, specifically within the striatum, has been implicated in TS. Imaging studies have identified a reduction in the volume of the striatum in TS patients (Peterson et al., 2003; Bloch et al., 2005) and post mortem studies indicate that this loss can primarily be attributed to a specific reduction in the number of striatal GABAergic interneurons (Kalanithi et al., 2005; Kataoka et al., 2010). Functional imaging studies in TS patients have also found a widespread GABAergic dysfunction in multiple BG nuclei, including the striatum (Lerner et al., 2012).

Disruption of local GABA_A_ transmission within the striatum has been shown to induce abnormal movements or behavioral states in model animals (Worbe et al., 2009). Early studies found that local administration of GABA_A_ antagonists, such as picrotoxin or bicuculline into the striatum of rats (Marsden et al., 1975; Tarsy et al., 1978) and monkeys (Crossman, 1987) induces repetitive contralateral jerk-like movements which were initially termed dyskinesia or myoclonus. More recent studies in monkeys suggested that these movements can be used to model the motor tics observed in TS (McCairn et al., 2009; Worbe et al., 2012; Bronfeld et al., 2013). In the current study we sought to characterize the abnormal movements induced by striatal disinhibition in the rat, using similar tools and analyses to the research performed on primates. We used local microinjections of bicuculline into the anterior and posterior parts of the rat striatum and examined the induced abnormal movements and behaviors, their progression in time, and their somatic distribution.

## Materials and methods

### Animals

Twenty-eight adult male Sprague Dawley rats weighing 316–460 g (375 ± 40 g, mean ± STD) were used in this study. Rats were maintained under conditions of controlled temperature and humidity, in a 12:12 h light/dark cycle, with free access to food and water. Data from three male Macaca Fascicularis monkeys was used in this study. All primate procedures were previously described in detail (McCairn et al., 2009; Bronfeld et al., 2011). All procedures were approved and supervised by the Institutional Animal Care and Use Committee (IACUC) and were in accordance with the National Institute of Health Guide for the Care and Use of Laboratory Animals and the Bar-Ilan University Guidelines for the Use and Care of Laboratory Animals in Research. This study was approved by the National Committee for Experiments in Laboratory Animals at the Ministry of Health (permit number 6–01–11).

### Surgical procedure

**Figure 1:**
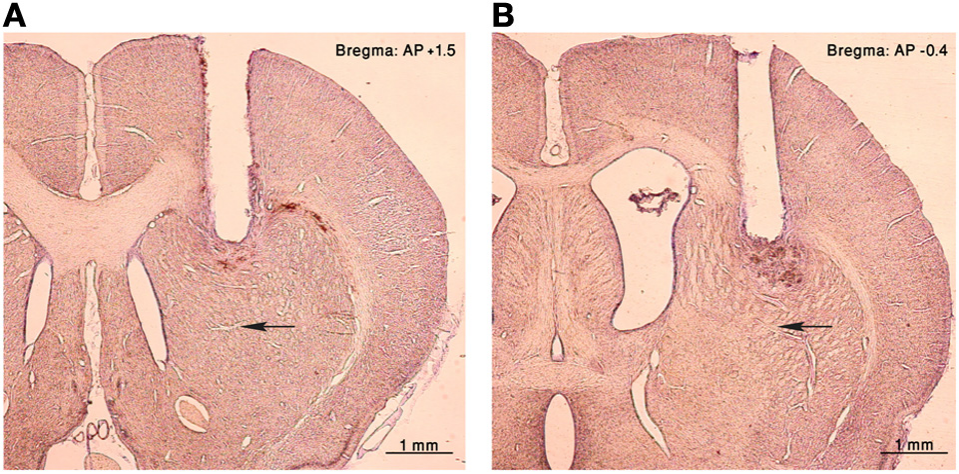
**Histology**. Nissl staining of coronal sections of the right hemisphere from an injected rat. A cannula lesion can be seen in the **(A)** anterior and **(B)** posterior striatum, black arrows point to the corresponding injection sites.

Rats were sedated with 5% isoflurane and then injected intra-peritoneally with ketamine HCl (100 mg/kg) and xylazine HCl (10 mg/kg). Anesthesia was maintained using a mixture of isoflurane 0.5–1% and oxygen and supplementary injections of ketamine were administered as required. The rat's head was fixed in a stereotaxic frame (Stoelting Co., Wood Dale, IL, USA). After sterilization of the skin and local infiltration with lidocaine (10 mg/ml) to reduce pain, an incision was made in order to expose the skull surface. Connective tissue was removed and the skull surface cleaned. Holes were drilled bilaterally in the skull targeting the anterior (AP: + 1.5, ML: ±2.5, DV: 3) or posterior (AP: −0.4 to −0.5, ML: ±3.5, DV: 3) striatum (Figure 1) (Paxinos and Watson, 2007). Guide cannulae (stainless steel 25 G tubes) were inserted to a location 2 mm above the injection target and sealed with a cannula-dummy (stainless steel wire 28 G). Most animals were implanted in both hemispheres with only one cannula in each hemisphere, but in a subset of animals (4 rats) two cannulae were implanted in each hemisphere. The implantation was secured to the skull with screws and dental acrylic cement (Coltene/Whaledent Inc., Cuyahoga Falls, OH, USA). Norocarp (Carprofen, 4 mg/kg) was injected subcutaneously post-surgery to relieve pain. Experimental sessions began after the animals recovered from surgery (typically 7–10 days).

### Microinjections

We used microinjections of bicuculline, which is a competitive GABA_A_ antagonist which can also inhibit SK channels (Stocker et al., 1999). Bicuculline methiodide (Sigma-Aldrich, Rehovot, Israel) was dissolved in physiological saline or artificial cerebrospinal fluid (aCSF containing (in mM): 145 NaCl, 15 Hepes, 2.5 KCl, 2 MgCl_2_, 1.2 CaCl_2_, PH 7.4 with NaOH) to a final concentration of 1 μg/μl. A volume of 0.5–1 μl was pressure injected at a rate of 0.35 or 0.5 μl/min (NE-1000, New Era Pump Systems, Farmingdale, NY, USA) using a 10 μl syringe (Hamilton, Reno, NV, USA). The injection was applied through an injection cannula (stainless steel 30 G tube) which was attached to the syringe via a flexible tube (Tygon microbore tube, Component Supply Company, Fort Meade, FL, USA). The injection cannula was manually inserted into the guide cannula which was fixed in the acrylic, with its tip located 2 mm past the tip of the guide cannula in the striatum.

### Experimental sessions

Experimental sessions began with a period of normal behavior (typically 10 min) within the behavioral observation cage, to establish a baseline behavior and allow the animals to acclimatize to the cage. Following this period the injection cannula was inserted and bicuculline was injected into the striatum over 1–3 min. The injection cannula was removed 1–2 min after the end of the injection, and the cannula-dummy was re-inserted. The animals' behavior was monitored continuously throughout the session and some sessions were filmed for detailed offline analysis using high speed video (two cameras at 50 frames/s each, uEye, IDS Imaging Development Systems, Obersulm, Germany) and acquired using StreamPix 4 (Norpix, Montreal, Canada). Sessions ended and the animals returned to their home cage at least 40 min after the microinjection (if no behavioral abnormalities were observed), or at least 10 min after bicuculline-induced behavioral abnormalities disappeared. Control injections of saline or aCSF were performed in some animals, using the same experimental design.

### Histology

The position of the microinjections was verified at the end of the experiments using histology. Animals were anesthetized and transcardially perfused with physiological saline followed by 10% paraformaldehyde. The brains were removed and immersed in a fixation solution of 30% sucrose and 10% paraformaldehyde for a minimum of 48 h. Brains were then frozen and sliced (50 μm coronal sections) using a cryostat microtome. Sections were mounted on glass slides, stained with Cresyl violet, and subsequently examined under a microscope to verify the location of the cannulae.

### Data analysis

Frame-by-frame analysis of sessions that were video recorded was used for all detailed temporal analyses of the tics. Tics were marked by the timing of the first frame in which the tic-related deflection of the relevant body part was observed. Each session was viewed from two angles (top and side) to ensure optimal identification of tic movements. Calculations of the average and the coefficient of variation (CV) of the inter-tic-intervals (ITIs) for both rats and monkeys were based on a representative video segment of 1–3 min from each session. All data analyses were performed using custom-written MATLAB code (MATLAB 2010A, MathWorks, Natick, MA, USA). All measures in the Results section are described as mean±STD unless stated otherwise.

## Results

Eighty-nine microinjections were performed, 53 in the anterior (28 cannulae) and 36 in the posterior (23 cannulae) striatum (Figure 1). One to five injections were performed in each cannula, and repeated injections typically induced similar behavioral effects. Motor tics were induced in 36/53 (68%) anterior and 21/36 (58%) posterior injections. Tics always appeared in the contralateral side to the injection and none were observed in the ipsilateral side. Tics were characterized as repetitive brief muscle contractions in an isolated muscle group of the hindlimb, forelimb, or face (see supplementary video). Tic-related movements ranged from small twitches of one finger to strong deflections of an entire limb or even the torso. During the course of a single session, small tics would initially be observed which would rapidly increase in amplitude and become more pronounced, usually maintained a stereotypic shape and size throughout the session, until they gradually decreased and reached complete cessation.

**Figure 2:**
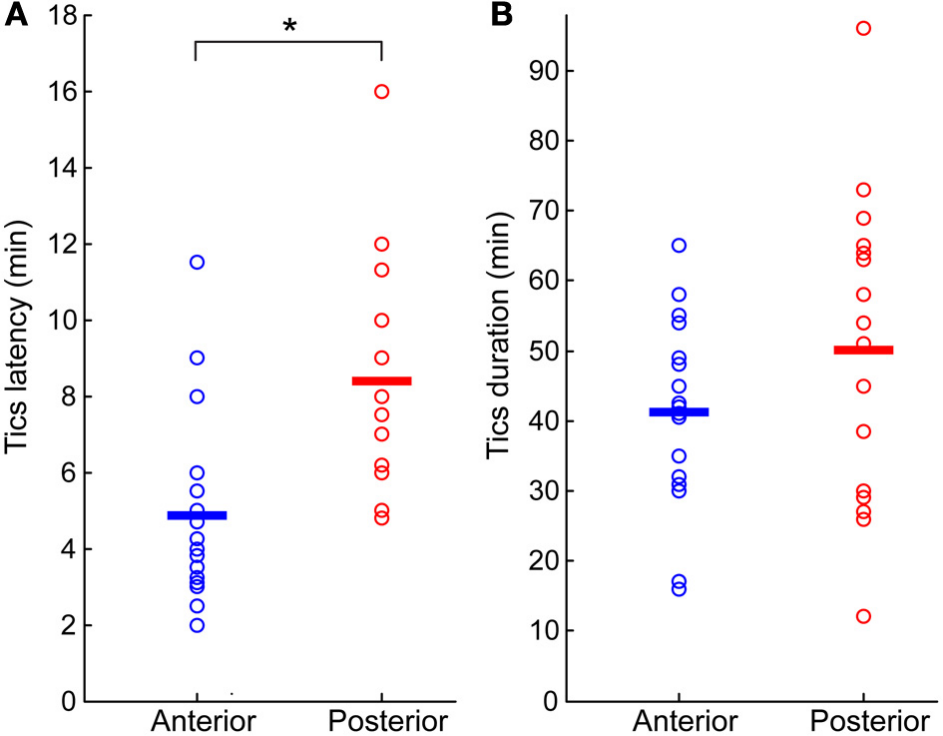
**Bicuculline-induced motor tics period**. **(A)** Latency to tic onset (time difference between microinjection onset and the appearance of the first motor tic). **(B)** Duration of tic expression (time difference between the first and last observed tics). In both graphs, results are shown separately for sessions in which the microinjection was targeted to the anterior (blue marker) or posterior (red marker) striatum. Horizontal lines indicate the group mean. ^*^p < 0.01.

**Figure 3:**
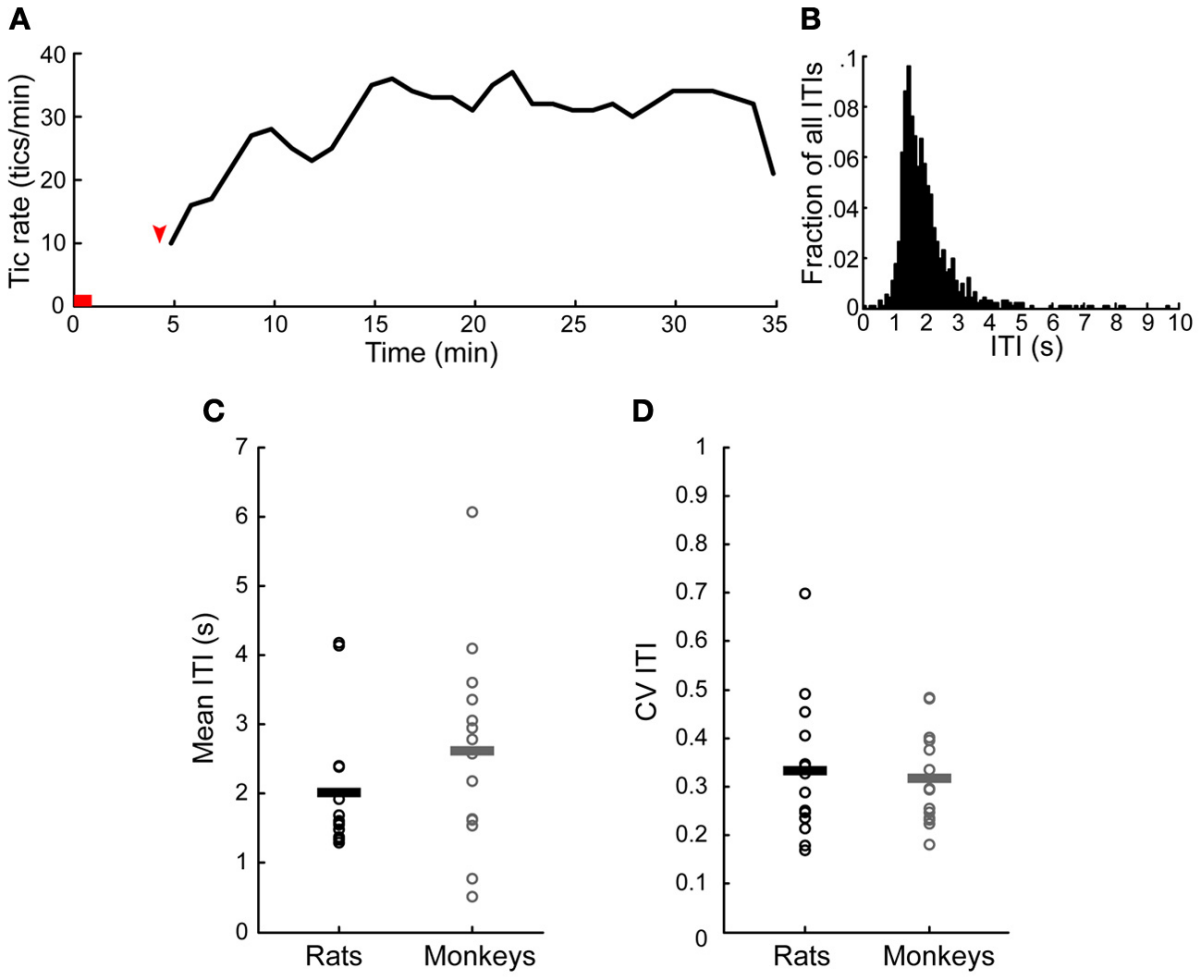
**Temporal properties of motor tics**. **(A)** An example of tic rate of an entire session (injection to the anterior striatum which induced forelimb tics). Horizontal red line indicates the injection time. Red arrow indicates time of first tic. **(B)** Histogram of the inter-tic intervals (ITIs) taken from the session presented in **(A)**. **(C,D)** A comparison of the **(C)** mean and **(D)** coefficient of variation (CV) of the ITIs observed in monkeys and rats throughout all the sessions. Horizontal lines indicate the group mean.

Tics appeared within 6 ± 3 min from the onset of the microinjection. However, the tic latency was significantly shorter following injections to the anterior than to the posterior striatum (latency anterior 4.8 ± 2.3 min, latency posterior 8.4 ± 2.8 min, *p* < 0.001, two-tailed *t*-test) (Figure 2A). Tics lasted for an average of 45 ± 18 min, with no significant difference between anterior and posterior locations (duration anterior 41 ± 13 min, duration posterior 50 ± 22 min, *p* > 0.1, two-tailed *t*-test) (Figure 2B). During the course of a session tic frequency varied around a mean base frequency which was typical to that session (Figure 3A). Across animals, the mean ITI ranged between 1.3 and 4.1 s, with an average of 2.02 ± 0.97 s (*N* = 14 sessions). The ITIs observed in rats were not significantly different from those observed in monkeys following striatal disinhibition, for which the mean ITI ranged between 0.5 and 6 s, with an average of 2.6 ± 1.44 (Figure 3C, *N* = 14 sessions, Mann–Whitney *U*-test, *p* > 0.1). In both monkeys and rats the length of ITIs was irregularly distributed (Figure 3B), as was evident by their low *CV* values (Figure 3D, rats *CV* = 0.33 ± 0.14, monkey *CV* = 0.32 ± 0.1, Mann–Whitney *U*-test, *p* > 0.1).

**Figure 4:**
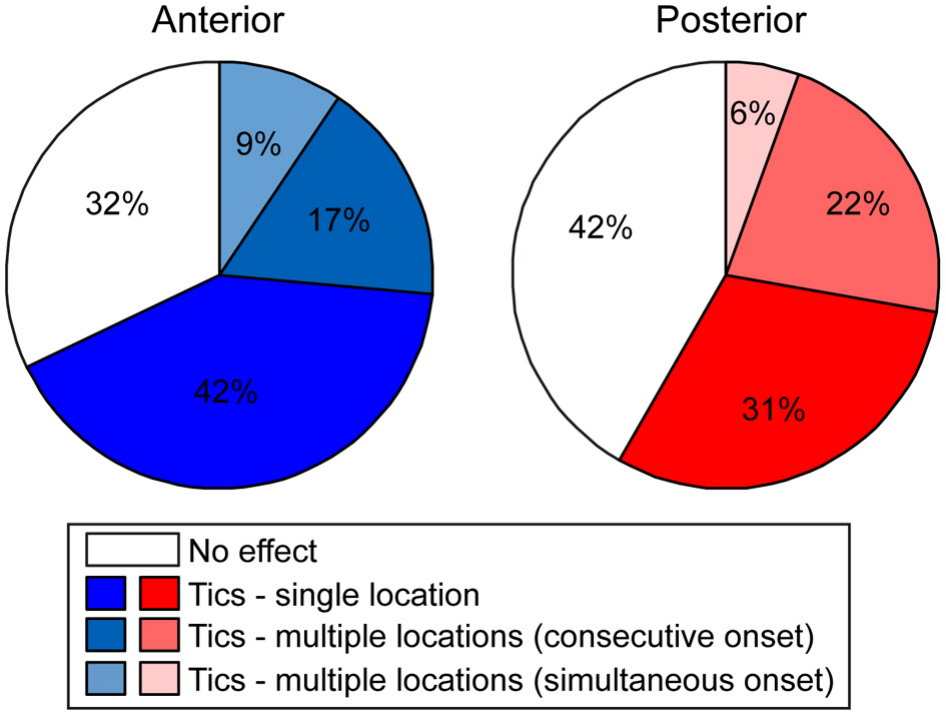
**Tic somatic localization**. Behavioral effects of bicuculline microinjections into the anterior (left) or posterior (right) striatum, classified according to the extent of localization of motor tics.

Following microinjection, tics were usually observed in a single body part and were limited to this location throughout the session [22/36 (61%) anterior and 11/21 (52%) posterior tic-inducing injections]. However, sometimes [9/36 (25%) anterior injections, 8/21 (38%) posterior injections] tics appeared in an additional location later during the session; e.g., tics observed initially only in the hindlimb progressed later to the forelimb, in which case tics were expressed simultaneously in both locations. In a minority of cases [5/36 (14%) anterior injections, 2/21 (10%) posterior injections] the tics appeared in two locations at once (Figure 4).

**Figure 5:**
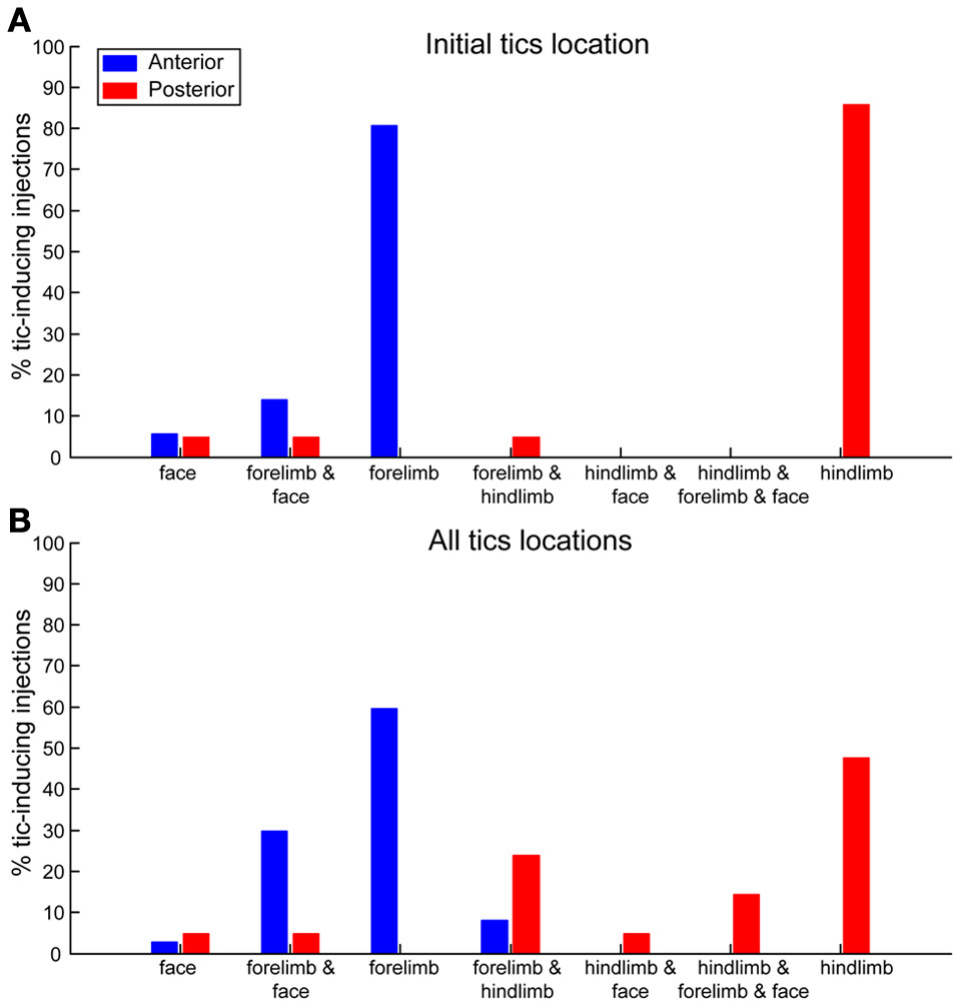
**Somatotopic organization of tics**. Percentages of bicuculline microinjections into the anterior (blue) or posterior (red) striatum inducing tics in different body parts. **(A)** Body part in which the first tics following microinjection were observed. **(B)** All body parts in which tics were observed at any stage during the session.

The location of the tics followed a somatotopic organization relative to the injection site such that forelimb tics were more commonly induced by injections to the anterior striatum, whereas hindlimb tics were mostly induced by injections to the posterior striatum. This effect was most apparent when examining the first location in which tics were expressed following microinjection (chi-square test with Yates' correction, χ^2^_(4)_ = 44.23, *p* < 0.001). In 29/36 (80%) anterior microinjections the tics were initially expressed in the contralateral forelimb. All other microinjections in the anterior location initially induced tics in the head/face muscles alone (2/36, 6%) or were simultaneous with forelimb tics (5/36, 14%). Hindlimb tics were never expressed as the first effect following anterior striatal microinjections (0/36, 0%). In contrast, posterior microinjections induced hindlimb tics with the shortest latency in 18/21 cases (85%). The first effects of the remainder of the posterior microinjections were tics expressed either in head/face muscles alone, in the face and forelimb areas together, or in the hindlimb and forelimb simultaneously [1/21 (5%) injections each case] (Figure 5A). This somatotopic organization was maintained even when examining all locations in which tics were expressed throughout the session [chi-square test with Yates' correction, χ^2^_(6)_ = 32.59, *p* < 0.001]. Forelimb tics were almost always (35/36, 97%) observed following injections into the anterior striatum. Forelimb tics induced by anterior injections were most commonly expressed alone [21/36 (58%)] or in conjunction with head/face tics [11/36 (31%) injections], and only seldom together with hindlimb tics (3/36, 8%). Hindlimb tics were the most common effect of injections into the posterior striatum (19/21, 90%). Hindlimb tics were expressed alone [10/21 (48%) injections] or in combination with forelimb tics (5/21, 24%) facial tics (1/21, 5%) or both (3/21, 14%). Only 2/21 (10%) posterior injections did not induce hindlimb tics, but rather tics in head/face muscles alone (1/21, 5%) or together with forelimb tics (1/21, 5%) (Figure 5B).

To control for the possible effects of cortical damage on tic expression we implanted a subset of animals with two cannulae in each hemisphere, over both the anterior and the posterior striatum. Overall, the presence of two cannulae in each hemisphere did not affect the somatotopic distribution of the tics. In all animals with two cannulae per hemisphere, the first location in which tics were expressed was the contralateral forelimb following anterior injections (*N* = 11 injections) and the contralateral hindlimb following posterior injections (*N* = 5 injections). The somatotopic distribution of all tic locations per session (Table 1) also did not significantly differ between animals with one or two cannulae per hemisphere, for either the anterior (chi-square test with Yates' correction, χ^2^_(3)_ = 5.6, *p* > 0.1) or the posterior (chi-square test with Yates' correction, χ^2^_(5)_ = 1.33, *p* > 0.1) injections.

**Table 1:** **Comparison of tics somatic distribution between animals with one or two cannulae implanted in each hemisphere**.

**No. of Cannulae**	**Face**	**Forelimb and face**	**Forelimb**	**Forelimb and hindlimb**	**Hindlimb and face**	**Hindlimb, forelimb and face**	**Hindlimb**
**ANTERIOR**							
2 cannulae (*N* = 11 injections)	0% (0)	9% (1)	64% (7)	27% (3)	0% (0)	0% (0)	0% (0)
1 cannula (*N* = 25 injections)	4% (1)	40% (10)	56% (14)	0% (0)	0% (0)	0% (0)	0% (0)
**POSTERIOR**							
2 cannula (*N* = 5 injections)	0% (0)	0% (0)	0% (0)	20% (1)	0% (0)	20% (1)	60% (3)
1 cannula (*N* = 16 injections)	6% (1)	6% (1)	0% (0)	25% (4)	6% (1)	12% (2)	45% (7)

Additional hyper-behavioral abnormalities were observed after some of the striatal bicuculline microinjections. These included hyperactivity, expressed as increased locomotion with frequent switches between different behaviors from the rat's normal behavioral repertoire (e.g., walking, rearing, sniffing, eating, etc.), circling around the cage perimeter due to an increased tendency to turn in one direction (contraversive to the injected hemisphere) and pivoting—on-the-spot rotations to the contralateral side to the injection. Each of these behaviors could be observed alone or in combination with another, and their expression, latency and duration were independent of the expression of tics. Hyper-behavioral abnormalities were more commonly observed following injections to the posterior [19/36 (53%) injections] than the anterior [13/53 (25%) injections] striatum [chi-square independence test, χ^2^_(1)_ = 7.43, *p* < 0.01]. Such behaviors could be observed both following tic-inducing microinjections [10/36 (28%) anterior injections, 10/21 (48%) posterior injections] and microinjections that did not induce tics [3/17 (18%) anterior injections, 9/15 (60%) posterior injections]. Another phenomenon which was sometimes observed following bicuculline microinjection was an abnormal posture of the limb. This abnormality was observed only in the hindlimbs following injections to the posterior striatum, usually in relation to hindlimb tics: at the end of a tic, the leg would not return to its normal position but rather maintained an abnormal posture. An abnormal posture was observed in 11/21 (52%) posterior injections in which it waxed and waned during the course of a session, such that it was evident only during some but not all of the tics.

In many sessions [23/36 (64%) anterior and 16/21 (76%) posterior tic-inducing injections] tetanic episodes were sporadically interspersed within the tic train. These episodes included several seconds in which the animal expressed multiple high-frequency successive tics, and would stop all other activities. The prevalence of these episodes varied widely across animals and sessions and ranged from once or a few times up to multiple times throughout the session.

Control injections of saline or aCSF were performed only in cannulae in which bicuculline previously induced the expression of tics: 7 anterior cases and 4 posterior cases. No abnormal behaviors or movements of any kind were observed following any of the control microinjections.

## Discussion

In the current study we used localized striatal microinjections of the GABA_A_ antagonist bicuculline to induce motor tics in freely behaving rats and characterized the nature and course of the induced abnormal movements and behaviors, most notably motor tics. Tics started within a few minutes following microinjection and their expression was mostly confined to a single body part. Tic expression followed a somatotopic organization, in which injections in the anterior striatum mainly induced tics in the contralateral forelimb, whereas injections to the posterior striatum induced hindlimb tics. The tics lasted for slightly under 1 h on average, and their frequency varied within and between different sessions and animals in the range of 15–45 tics per minute. In some cases, striatal bicuculline microinjections elicited other hyper-behavioral abnormalities, including general hyperactivity and contraversive circling or pivoting, as well as tetanic episodes which were interspersed within the tic trains.

Overall, motor tics induced by striatal disinhibition in rats demonstrated the main properties of tics induced in monkeys using a similar manipulation (Crossman et al., 1988; McCairn et al., 2009; Bronfeld et al., 2011): (1) In both species tics started within a few minutes following microinjection and were expressed as brief jerk-like movements manifested as retraction or contraction of the affected body part or muscle group on the contralateral side to the injection (Tarsy et al., 1978; Patel and Slater, 1987; Crossman et al., 1988; McCairn et al., 2009); (2) Tics were mostly focal in nature, but were sometimes expressed in more than one location (Crossman et al., 1988; McCairn et al., 2009; Bronfeld et al., 2011). Even in such cases, the tics remained isolated and did not resemble seizure-like whole body phenomena; (3) During tic expression the animals seemed to maintain normal motor and cognitive functions, as was evidenced by sustained exploration, rearing, grooming and eating in the rats, and by continued performance of a motor task by the monkeys (McCairn et al., 2009; Worbe et al., 2009); (4) Normal behavior was only paused during the brief tetanic episodes, which were observed in both monkeys and rats (McKenzie and Viik, 1975; Tarsy et al., 1978; McCairn et al., 2009); (5) The frequency of tics displayed variability during the course of a session as well as across different sessions and animals. However, the overall frequency range and the general temporal structure of tics throughout a session were remarkably similar between the species (McKenzie and Viik, 1975; Tarsy et al., 1978; McCairn et al., 2009). (6) The additional hyper-behavioral symptoms (hyperactivity and contraversive circling) which sometimes followed microinjections were common to both species. Such behaviors have been observed in both monkeys (Grabli et al., 2004; Worbe et al., 2009; Bronfeld et al., 2010) and rats (Wisniecki et al., 2003; Ikeda et al., 2010) following microinjections of GABA_A_ antagonists into the striatum or into an adjacent BG nucleus—the GPe. In the current study, hyperbehavioral symptoms were more common following microinjections into the posterior striatum, a location which is closer to the striatum-GPe border, compared with the anterior striatum location. This suggests that these symptoms may be mediated, at least in part, by diffusion of the bicuculline to the GPe, rather than by its direct effect in the striatum. Hindlimb dystonia was also sometimes observed following striatal bicuculline microinjections in the rats but not in monkeys. However, this phenomenon is not unique to the rats, as it was previously described in cats following striatal disinhibition (Yoshida et al., 1991; Yamada et al., 1995).

The main difference between motor tics induced by striatal disinhibition in monkeys and rats pertains to the somatotopic organization observed in the rat which was not detected in the monkey (Crossman et al., 1988; McCairn et al., 2009; Worbe et al., 2009, 2013; Bronfeld et al., 2011). Somatotopic organization of tic-inducing striatal disinhibition in the rat has been observed in previous studies. It is evident both from a combination of different studies, each described injections in a different striatal location which induced tics in different body parts (McKenzie et al., 1972; Patel and Slater, 1987; Muramatsu et al., 1990; Nakamura et al., 1990) as well as from a study that explicitly compared the effects of injections in different striatal loci (Tarsy et al., 1978). Overall, the somatotopic organization followed an anterior-posterior axis, in which tics were observed in orofacial muscles following injections into the anterior striatum (Nakamura et al., 1990), in the forelimbs following injections into the central striatum (Tarsy et al., 1978; Patel and Slater, 1987) and in the hindlimbs following injections into the posterior striatum (Tarsy et al., 1978). Mixed effects were also observed, mostly following the same somatotopic organization (head and forelimb tics, forelimb and hindlimb tics). We also observed some degree of co-expression of tics in multiple body parts, which may be attributed either to small variability in inter-animal injection sites or to diffusion of the injected substance across multiple somatotopic territories (Yoshida et al., 1991).

A possible explanation for the different effects of differentially located injections is the somatotopic organization of the striatum. Although there is a general consensus that the striatal motor territory is somatotopically organized, as evidenced by anatomical connectivity (Brown, 1992; Ebrahimi et al., 1992; Brown et al., 1998), neuronal activity (Carelli and West, 1991), metabolic mapping (Brown and Sharp, 1995) and lesion studies (Pisa and Schranz, 1988), findings tend to differ regarding the topographical arrangement of this organization. Early studies reported that cortical fibers projecting to the striatum are topographically well-organized along an anterior-posterior axis, creating a somatotopic map in which the head, forelimb, and hindlimb are represented in successively posterior striatal locations (Webster, 1961). More recent studies suggest a more complex organization of cortico-striatal connectivity, in which cortical projections cluster in partially overlapping groups and create a three dimensional complex map along the anterior-posterior, ventral-medial and dorso-ventral axes (Carelli and West, 1991; Brown, 1992; Ebrahimi et al., 1992; Brown et al., 1998). The effects of local striatal disinhibition in our study and in previous ones (McKenzie et al., 1972; Tarsy et al., 1978; Patel and Slater, 1987; Muramatsu et al., 1990; Nakamura et al., 1990) support the former organization but not the latter.

An alternative explanation for the observed somatotopy is that the effects of local striatal disinhibition might be mediated by and in fact dependent on the involvement of the cerebral cortex. Cortical involvement was suggested by Tarsy and colleagues, who found that when the injection cannula was inserted into the rat striatum at an angled trajectory that spared the sensorimotor cortex overlaying the striatum, tics did not appear following the injection of GABA antagonists (Tarsy et al., 1978). Remarkably, tics could be elicited by a combination of striatal disinhibition via an angled cannula combined with cortical damage (done by merely inserting a second cannula into the cortex). These findings were later challenged by Patel and colleagues who found that cortical damage had no effect on the manifestation of tics since they were equally induced by straight (passing through the cortex) and angled (leaving the cortex intact) striatal microinjections (Patel and Slater, 1987). In the current study, we had a subset of animals which had simultaneous cortical damage in the areas overlaying both the anterior and posterior striatal injection sites. The somatic distribution of tics in these animals was similar to the distribution observed in animals implanted with only one cannula in each hemisphere, which suggests that the cortical damage has little or no effect over the determination of tic locations.

In the primate, the somatotopic organization of the striatum has been well-established by converging results of anatomical studies (Kunzle, 1975; Flaherty and Graybiel, 1991; Takada et al., 1998), recordings of neuronal activity (Nambu et al., 2002) and electrical microstimulation (Alexander and DeLong, 1985). Overall it follows a dorsal-ventral organization, in which the hindlimbs are represented at the most dorsal aspect of the striatum, followed by the forelimb and the head/face regions which are represented at gradually more ventral parts. However, the effects of striatal disinhibition fail to display any somatotopic organization. Instead, tics are expressed predominantly in orofacial muscles, and to a lesser extent in forelimb muscles (Crossman et al., 1988; McCairn et al., 2009; Worbe et al., 2009, 2013; Bronfeld et al., 2011) and rarely in hindlimb muscles (Crossman et al., 1988), regardless of the striatal injection site. The basis of this inter-species difference and the lack of tic-related somatotopy in monkeys are unclear. A possible explanation might be related to an over-representation of orofacial regions in the primate striatum (Miyachi et al., 2006). This is in line with tic expression in human TS patients, in which head and face tics are typically the first and most common symptom of the disorder (Kurlan, 2004).

Previous studies describing the behavioral outcomes of striatal disinhibition referred to the induced abnormal movements as choreiform (McKenzie and Viik, 1975), dyskinesia (Muramatsu et al., 1990) or more commonly, myoclonus (Pycock et al., 1976; Tarsy et al., 1978; Patel and Slater, 1987; Crossman et al., 1988). However, recent studies in monkeys suggest that this model may be applicable to the study of motor tics (McCairn et al., 2009; Bronfeld et al., 2011, 2013; Bronfeld and Bar-Gad, 2013; Worbe et al., 2013). The results of the current study suggest that striatal disinhibition in rats may also be used as a model for tics. The isolated, brief, and sudden nature of the abnormal movements in rats is qualitatively more similar to tics rather than chorea, which tends to consist of smooth continuous movements (Mark, 2004). Several other characteristics of these movements, including their spontaneous nature, their persistence both during rest and active movements, their irregular timing and their localized expression are more consistent with the clinical expression of tics rather than myoclonus (Obeso et al., 1985; Shibasaki and Hallett, 2005; Vercueil, 2006). Finally, while tics have been associated with dysfunction of the CBG system (Bronfeld and Bar-Gad, 2013), myoclonus is attributed to other neuronal structures (Caviness and Brown, 2004; Shibasaki and Hallett, 2005; Cassim and Houdayer, 2006). Another feature of the striatal disinhibition model is the occurrence of other hyperbehavioral disorders in both monkeys and rats. These behavioral abnormalities are reminiscent of attention deficits, hyperactivity and repetitive/compulsive behaviors (Grabli et al., 2004; Worbe et al., 2009) which are often observed in TS patients (Freeman et al., 2000) but not in myoclonus patients. This comorbidity in both humans and in the animal model both strengthens the validity of the model and suggests that a common neurophysiological mechanism may underlie these different disorders (Bronfeld and Bar-Gad, 2013; Bronfeld et al., 2013).

Multiple neuronal sub-populations and pathways within the striatum might mediate the tic-inducing effects of bicuculline. These include the GABA_A_-mediated collateral connections between MSNs (Tunstall et al., 2002), connections between striatal interneurons and MSNs (Koos and Tepper, 1999), interactions between different types of interneurons leading to indirect modulation of the MSNs (English et al., 2012) and feedback afferents from the GPe (Bevan et al., 1998; Mallet et al., 2012). A recent study demonstrated that a selective inhibition of one type of striatal interneurons (FSI) could induce complex abnormal movements in mice, including dystonic postures and jerking movements (Gittis et al., 2011). These findings are in line with anatomical observations of selective loss of FSIs in human TS patients (Kalanithi et al., 2005). However, FSI dysfunction does not account for the complete behavioral manifestation of motor tics, and further studies are required to identify the neural substrates and interactions involved in the generation of bicuculline-induced motor tics.

The resemblance between striatal disinhibition in monkeys and rats, and indeed between these animal models and the clinical symptoms of human TS patients, suggests that rats may be a valid animal species for the continued exploration of tics through this model. A rat-based model for TS holds great promise for advancing research in this field. The phenomenological similarities of tics induced in animals and those observed in human patients offer a unique opportunity to directly study the physiological and neurological mechanisms underlying and associated with motor tics. Furthermore, this model may used as a novel platform for the investigation of novel therapeutic targets of TS, including screening of pharmacological agents, testing novel medical devices and behavioral manipulations.

### Conflict of interest statement

The authors declare that the research was conducted in the absence of any commercial or financial relationships that could be construed as a potential conflict of interest.

## Abbreviations

BG: basal ganglia
CBG: cortico-basal ganglia
ITI: inter-tic-interval
MSN: medium spiny neuron
TS: Tourette syndrome.
